# Mutational Analysis of the High-Affinity Zinc Binding Site Validates a Refined Human Dopamine Transporter Homology Model

**DOI:** 10.1371/journal.pcbi.1002909

**Published:** 2013-02-21

**Authors:** Thomas Stockner, Therese R. Montgomery, Oliver Kudlacek, Rene Weissensteiner, Gerhard F. Ecker, Michael Freissmuth, Harald H. Sitte

**Affiliations:** 1 Center of Physiology and Pharmacology, Institute of Pharmacology, Medical University Vienna, Vienna, Austria; 2 Department of Medicinal Chemistry, University of Vienna, Vienna, Austria; University of Calgary, Canada

## Abstract

The high-resolution crystal structure of the leucine transporter (LeuT) is frequently used as a template for homology models of the dopamine transporter (DAT). Although similar in structure, DAT differs considerably from LeuT in a number of ways: (i) when compared to LeuT, DAT has very long intracellular amino and carboxyl termini; (ii) LeuT and DAT share a rather low overall sequence identity (22%) and (iii) the extracellular loop 2 (EL2) of DAT is substantially longer than that of LeuT. Extracellular zinc binds to DAT and restricts the transporter‚s movement through the conformational cycle, thereby resulting in a decrease in substrate uptake. Residue H293 in EL2 praticipates in zinc binding and must be modelled correctly to allow for a full understanding of its effects. We exploited the high-affinity zinc binding site endogenously present in DAT to create a model of the complete transmemberane domain of DAT. The zinc binding site provided a DAT-specific molecular ruler for calibration of the model. Our DAT model places EL2 at the transporter lipid interface in the vicinity of the zinc binding site. Based on the model, D206 was predicted to represent a fourth co-ordinating residue, in addition to the three previously described zinc binding residues H193, H375 and E396. This prediction was confirmed by mutagenesis: substitution of D206 by lysine and cysteine affected the inhibitory potency of zinc and the maximum inhibition exerted by zinc, respectively. Conversely, the structural changes observed in the model allowed for rationalizing the zinc-dependent regulation of DAT: upon binding, zinc stabilizes the outward-facing state, because its first coordination shell can only be completed in this conformation. Thus, the model provides a validated solution to the long extracellular loop and may be useful to address other aspects of the transport cycle.

## Author Summary

The dopamine transporter (DAT) regulates dopaminergic neurotransmission in the brain and is implicated in numerous human disease states. DAT is unique among the monoamine neurotransmitter transporter family because its substrate transport is inhibited by extracellular zinc. DAT homology models rely upon the crystal structure of LeuT solved in 2005. LeuT and DAT share a relatively low overall sequence identity of 22%. In addition, the length of the second extracellular loop of DAT exceeds that of LeuT by 21 residues. The zinc binding site cannot be directly modeled from the LeuT template alone because of these differences. Current available homology models of DAT focused on substrate or inhibitor binding rather than on the second extracellular loop. We exploited the specificity of the zinc binding site to build and calibrate a DAT homology model of the complete transmembrane domain. Our model predicted that the zinc binding site in DAT consists of four zinc co-ordinating residues rather than three that had been previously identified. We verified this hypothesis by site-directed mutagenesis and uptake inhibition studies.

## Introduction

The dopamine transporter (DAT) is a member of the neurotransmitter∶sodium symporter family [1]. DAT actively removes dopamine (DA) from the synaptic cleft by re-uptake into the presynaptic neuron, utilizing the electrochemical sodium gradient. Thus, DAT is a key regulator of the spatial and temporal extraneuronal DA concentration [2]. Human DAT is of particular clinical relevance because dopaminergic transmission plays a key role in several disease entities, e.g. schizophrenia, Parkinson's disease, addiction and drug dependence. Illicit drugs which target DAT, such as cocaine and amphetamine, are among the most commonly abused drugs worldwide. Accordingly, the molecular mechanisms by which DAT operates are a subject of intense scientific and public interest [3].

Translocation of a hydrophilic substrate across the lipid bilayer has been conceptualized by a theoretical framework that posited an alternating access mechanism [4]. This model is supported by a number of recent crystallization studies that provided snapshots of various conformations of a NSS homologue from the thermophilic bacterium *Aquifex aeolicus* (LeuT) [5]–[7]. The crystals showed a movement of the bundle domain (consisting of helices 1, 2, 6 and 7) relative to the scaffolding domain [8]–[10], of which the core helices 3, 4, 8 and 9 are arranged in a hash-like shape. Accordingly, this internal scaffold is also referred to as hash domain. The bundle domain rotates by 30–40 degrees during the transition from the open-outward facing to the inward-facing conformation [5]. Although the substrate binding pocket shows a sequence identity of ∼50%, the overall sequence identity between LeuT and DAT is less than 25%. LeuT has also a much shorter second extracellular loop than DAT and lacks a critical Cl^−^ binding site, which is necesscary for DAT substrate translocation [11]–[14].

The relationship between DAT structure and function has been extensively studied using numerous strategies, including mutagenesis and chimeric domain swapping [15], selected cysteine accessibility studies [16] and by examination of a high-affinity zinc-binding site [17]. Extracellular zinc is a potent inhibitor of dopamine re-uptake in both, synaptosomes and heterologous expression systems with an IC50 in the low µM range [18], [19]. The zinc-binding site can serve as a DAT specific molecular ruler which defines the spatial micro-environment between the transmembrane helices 7, 8 and extracellular loop 2 (EL2). Three amino acid side chains have been identified in DAT which co-ordinate zinc: H193 in EL2, H375 in the first helical part of extracellular loop 4 (EL4A) and E396 in the second helix of extracellular loop 4 (EL4B) [18], [20]. The norepinephrine transport (NET) contains two out of the three zinc co-ordinating residues found in DAT, but transport catalyzed by NET is insensitive to zinc. If the third DAT zinc co-ordinating residue (H193) is grafted into NET (position 189), NET becomes susceptible to inhibition by zinc [18]. Similarily, the serotonin transporter (SERT) is also insensitive to inhibition by zinc [21], but can be rendered susceptible by appropriate mutations [22].

Extracellular zinc can reach concentrations of up to 10–30 µM in the brain under normal physiological conditions [23], [24]. Given the low µM IC50 values observed for zinc inhibition of dopamine uptake in *vitro*, there is strong evidence to suggest that zinc may be a physiologically important regulator of DAT function [25]. Importantly, extracellular zinc exerts effects on DAT function that go beyond mere uptake inhibition: zinc can also enhance amphetamine-induced, transporter-mediated release [21], [26] and DAT-mediated currents [26], [27].

Binding of zinc to the coordinating residues in the DAT (H193, H375 and E396) alters the conformational equilibrium between the inward- and outward-facing state of the DAT [28]. Thus, zinc constrains the movement of the DAT by selectively binding to one conformation. Interestingly, the crystal structures of LeuT in the inward- and outward-facing conformation reveal that the relative orientation of H375 and E396 changes during the transport cycle [5]. The third zinc co-ordinating residue, H193, is located in the EL2; thus, correct modeling of this loop is required to fully understand the effect of zinc on the transport cycle.

Modeling studies of human NSS transporters have focused on the transmembrane helices and the substrate binding site [29]–[43]. Although these studies usually included the EL2 loop, they did not report an analysis of the loop geometry, the disulfide bond or the spatial proximity with residues in the EL4. In addition, several molecular dynamics (MD) simulations of human NSS transporter have been carried out to date [36], [37], [41], [44]–[48]. Analysis of the conformations and properties of EL2 were often not reported. Huang et al. [37] developed a DAT model and studied dopamine and cocaine binding using MD simulations. They included knowledge on the disulfide bond and spatial proximity of residues H193, H375 and E396 in their model building process, but did not analyze the behavior of the EL2 in their simulations. Henry et al. [46] reported on a simulation of SERT which focused mainly on the co-transported ions. Gedeon et al [44] reported on 30 ns simulation studies of LeuT and DAT. They found stable transmembrane regions and observed in the DAT that the mobility of the EL2 loop exceeds that of the other loop regions by two to three fold. Koldso et al. [45] reported simulations of SERT in different conditions and observed a transition from the outward-facing to the inward-facing conformation; the authors reported per-residue mobility and found that the EL2 loop showed two to three times higher fluctuations than the other loops of the SERT.

Residue H193 should be close to H375 and E396 to allow zinc to be in contact with all three residues. Hence, we addressed the possible conformation of the EL2 loop and its repercussion for the transport cycle by using the zinc binding site of DAT as a molecular ruler to calibrate the resulting model. We present a refined model of the entire DAT transmembrane domain including EL2. The refined model was stable in a 200 ns long MD simulation. The mobility of the modeled EL2 was comparable to that of the other loops in DAT. Furthermore, our DAT model revealed that the zinc binding site consisted of four coordinating amino acid side chains rather than three that had been previously identified [18], [20], [49]. This prediction was confirmed by site-directed mutagenesis of D206 in EL2 thus providing an experimental validation of our refined DAT model.

## Results

**Figure 1:**
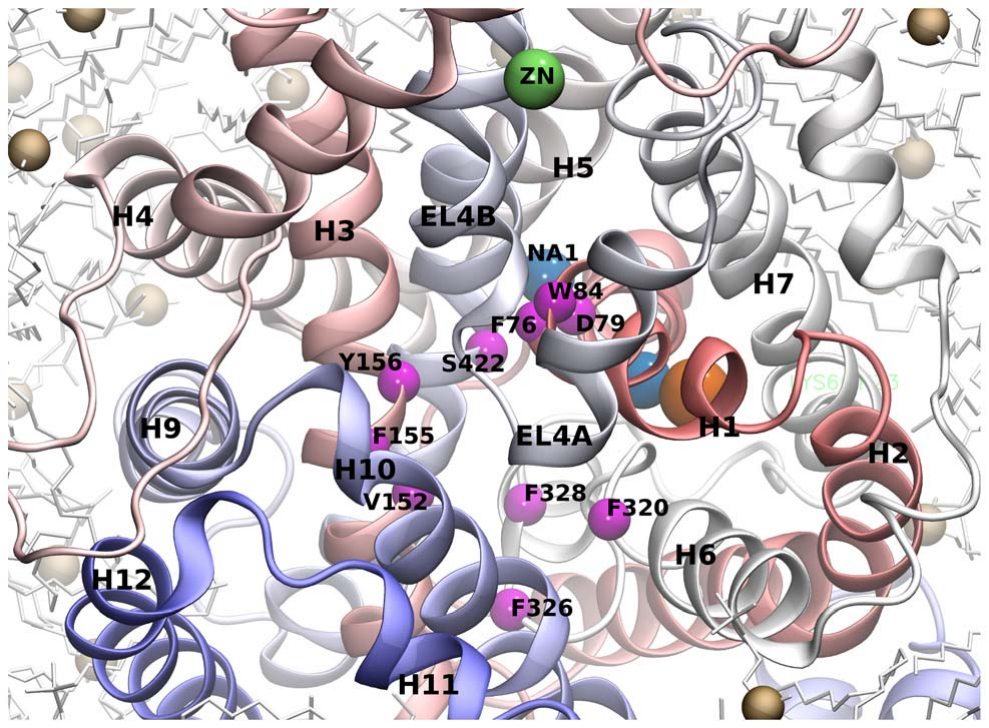
Mapping of residues known to interact with substrate. View into the outer vestibule of the membrane embedded DAT. Helices are color coded (N to C-terminus) from red to blue. The sodium ions are shown as blue spheres, the chloride ion in orange and the zinc ion in green. The Cα atoms of the vestibule lining residues known to interact with the substrate dopamine and the inhibitor cocaine are highlighted as magenta spheres.

An all-atom model of the complete transmembrane domain of DAT was developed in order to further elucidate the molecular basis of dopamine transport. This model was based on the leucine-bound outward-occluded high resolution LeuT crystal structure (PDB ID: 2A65) [7] as described in “materials and methods”. We first evaluated the compatibility of this model with published experimental findings. Several DAT mutations have previously been described and tested for their effects on binding and transport (for a comprehensive review see [50]). In particular, the residues lining the substrate and cocaine binding site have been extensively characterized, both experimentally [38], [51]–[54] and by molecular modeling [36]–[39], [47]. In line with previous data, we observed that these residues cluster in the outer vestibule and their side chains are oriented towards the pore (Fig. 1). According to site-directed mutagenesis studies, D79 is not only essential for substrate and cocaine binding but also for substrate transport [54]. In agreement with these findings, D79 interacted with sodium 1 and was oriented toward the S1 substrate-binding site in our model. Experimental evidence indicated that residue W84 could be in contact with bound cocaine, although this is still debated [38], [51], [53], [55]. We find W84 oriented towards the vestibule, delineating the vestibule border at the outer gate region that divides the S1 binding site from the cell exterior. The mutation of pore-oriented amino acid F155 in rat DAT, which lines the vestibule led to a stark increase in the K_m_ for [^3^H]DA uptake, and also decreased the affinity of [^3^H]CFT binding in DAT expressing COS cells [52]. In the same study, mutation of residue F319 in rat DAT (corresponding to residue F320 in human DAT) in helix 6 resulted in a significant decrease in [^3^H]DA uptake and [^3^H]CFT binding affinity. This residue was located on the opposing side of the vestibule and directly interacted with F155 in our model. Similarly, residue F76 on helix 1 and F325 (residue F326 in human DAT) on helix 6 line the S1 substrate binding site (Fig. 1); the corresponding mutations affect substrate uptake and cocaine binding [52]. Mutations of the residues V152, Y156, V328 and S422, which in our model also lined the S1 substrate binding site (Fig. 1), reduce both [^3^H]DA transport and [^3^H]CFT binding to DAT [38]. Furthermore, mutation of L80 located in helix 1, which interacted with Y156 in our model, also impairs substrate transport [38]. Thus, our model is consistent with the available experimental observations and similar to previously published models. Accordingly, this justifies the conclusion that the core of the transporter has been modeled correctly.

### Substrate permeation pathway

Similar to comparing the transporter core, we tested the validity of our DAT model with regard to the substrate permeation pathway, including (i) the salt bridge in the outer vestibule, (ii) the accessibility of residues within the vestibule, (iii) the salt bridge found at the inner gate, and (iv) the mutation of Y335, which impairs transport.

**Figure 2:**
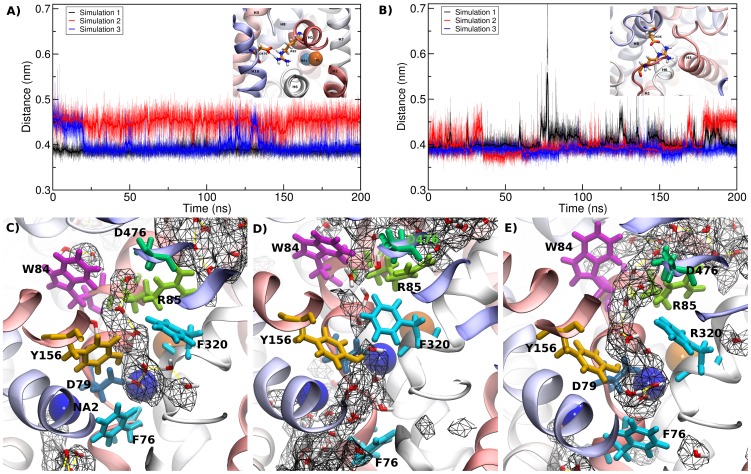
Salt bridges and water in the substrate permeation pathway. A) Time evolution of the salt bridge in the outer vestibule between R85 in transmembrane helix 1 and D476 in transmembrane helix 10. The structure of the salt bridge is shown in the insert. The distance is measured between the Cζ carbon of the guanidium group of R85 and the Cγ carbon of D476. B) Time evolution of the salt bridge between R60 and D436 in the inner vestibule. The structure of the salt bridge is shown in the insert. The distance is measured between the Cζ carbon of the guanidium group of R60 and the Cγ carbon of D436. C–E) A slice through the DAT showing an overlay of average water density (black hash) with a respective representative snapshot for simulation 1 (panel C), simulation 2 (panel D) and simulation 3 (panel E). Several residues in the S1 binding site and at the outer gate are shown for orientation.

A water mediated salt bridge has been reported to form between the conserved residues R85 and D476 (residue R30 and D404 in LeuT) in the occluded state of LeuT. However, in the open-outward conformation, the distance is increased between these two residues as the bundle domain rotates by ∼20 degrees relative to the hash domain [5], [9]. Our model was based upon the occluded state of LeuT. We found a stable ionic interaction between R85 and D476 as shown in Fig. 2A which was either mono-dentated of bi-dentated. We did not observe water molecules stably inserted as evident in the crystal structure of LeuT. The salt bridge stabilized the distance between H1 and H10 and thereby restricted the relative motion between hash and bundle domain.

We consistently found that water molecules occupied the S1 binding site, as indicated by the average water density shown as black grid in Fig. 2C–E. The water molecules in the S1 binding site form a continuous hydrogen bonded network that connects to the bulk solution in the extracellular space, which was occasionally interrupted: Fig. 2C–E displays snapshots from the simulations overlaid by the average water density. The constriction zone is formed by the outer gate, which we observed to consist of residues R85, D476, W84, F320, F155 and Y156. The salt bridge between R85 and D476 form the first layer followed by the hydrophobic layer consisting of W84, F320, F155 and Y156. We observed the charged side chain of D79 to be well hydrated. The sodium ions remained firmly bound to their binding site; they directly interacted with only one or two water molecules. However, no water density is visible next to the chloride ion. Interestingly, sodium ion 2 becomes increasingly hydrated from the cytosolic site in the second of the 200 ns simulations (Fig. 2D). Similar hydration from the cell interior was previously observed for SERT [45] and DAT [47]. Once fully hydrated, the sodium starts moving, as described below (in the next section).

Residue R60 has previously been shown to interact with D436 in DAT [56]. The mutation of either residue resulted in a marked decrease in [3H]DA uptake. The resulting mutant transporters are thought to reside in an inward-facing conformation. However, transport function of the R60A mutant was partially restored with zinc [57]. We found in all simulations that the side chains of these two residues formed very stable salt bridges (Fig. 2B). We observed only occasional opening for very brief time periods. Hence, their disruption would affect the stability of the outward-facing state.

Residue Y335 is also important for transport. The mutation of Y335 to alanine completely abolishes [^3^H]DA uptake [28] and shifts the transporter into a channel-like mode [27]. [3H]DA uptake is partially restored by the application of extracellular zinc, suggesting that this mutant is deficient in the return step to the outward-facing conformation [28]. We found Y335 to be located in the center of the inner vestibule. Water penetrated into the closed inner vestibule until it reached Y335, which suggests that this residue may play a dual role by (i) stabilizing the outward-facing conformation and (ii) forming the hydrophobic part of the inner gate.

### Ion binding

**Figure 3:**
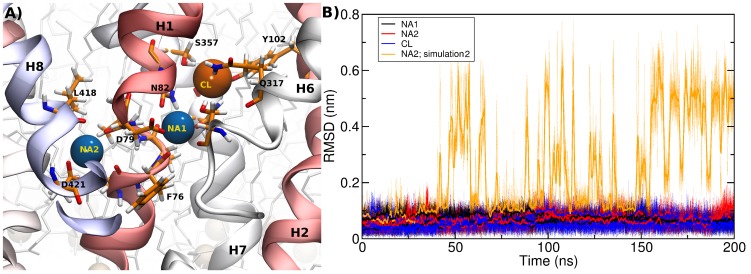
Co-transported ions. A) Representative snapshot showing the two bound sodium ions and the chloride ion. Residues interacting with the ions are highlighted. B) The ions are well bound to their respective binding sites, as the time evolution of the RMSD shows. The only exception is sodium 2 of simulation 2, which begins oscillating between its canonical position and a new position towards the intracellular side.

NSS transporters couple substrate transport to the ion gradients of both sodium and chloride. For example, the translocation of DAT substrates requires the sequential binding and co-transport of two sodium ions and one chloride ion. Both sodium binding sites are conserved between LeuT and DAT and have been modeled accordingly (Fig. 3A). Sodium 1 remained stably bound in all simulations, as shown in Fig. 3B. Sodium 1 interacted with and oriented the side chain of residue D79. This amino acid side chain interacts with the positively charged nitrogen of the monoamine substrates. Its mutation results in a non-functional transporter [54]. The residue corresponding to D79 is replaced with a glycine in all non-monoamine transporters, e.g. LeuT, GlyT and the GABA transporters. Substrates of these transporters are amino acids and thus supply a carboxyl group (absent in the monoamines substrates of DAT, NET and SERT) in *trans*. As observed in the LeuT crystal structures, this carboxyl group occupies the place of the side chain of D79 and directly interact with sodium 1. Sodium 2 remained stably bound only in two simulations, while it started moving towards the cytosol in the second 200 ns long simulation. We observed in the first 50 ns an increasing number of water molecules interacting with sodium 2 which began oscillating its position after 50 ns. The motion of the sodium was closely followed by the side chain of D421 in helix 8. In the next 100 ns, sodium 2 moved back and forth between its binding site and a position at the cytosolic oriented side of helix 8. The water file connecting the S1 binding site with the extracellular bulk solvent became interrupted during this period. We observed in the last 50 ns an increased preference for occupying the cytosolic site of helix 8. A similar behavior of sodium 2 was observed by Koldso et al [45] in simulations of SERT, but we did not observe complete opening of the R60-D436 salt bridge (R79-D425 in SERT) or transition to the inward-facing state.

Transport of substrates by NSS depends on chloride, while LeuT-mediated transport does not require chloride. The evidence that chloride is required for transport is strong, as transport is strongly reduced in its absence [12], [13], [58]. It is however still not fully established if the chloride gradient is necessary for co-transport of neurotransmitters. While the classical stoichiometry of DAT transport proposes that substrate transport is chloride dependent [59], Erreger et al. [58] have proposed that chloride plays a regulatory function, but its chemical gradient does not directly support transport, as they found that both internal and external chloride facilitate transport and that increasing intracellular chloride concentration did not affect transport associated currents. Previous modelling and mutational analyses [12], [13] located the chloride in a hydrophilic pocket. Consistent with this notion, we found the chloride atom stably bound in this pocket (Fig. 3B).

### Modeling of the EL2 loop

The entire EL2 consists of several segments: it begins with a loop, that includes the disulfide-bridge between C180 and C189 as well as H193 and is followed by the EL2-helix: this helix in the center of the extracellular loop is conserved from the procariotic LeuT to the human NSS transporter family. This structural element is followed by another loop element that connects the EL2-helix with transmembrane helix 4. LeuT serves as a template for both the EL2-helix and the second loop, while the first loop is in DAT by 21 residues longer than in the LeuT template. Our EL2 modeling focuses on creating this extended loop. Models of NSS transporters which include EL2 have already been published [31]–[33], [35]–[37], [39], [41], [42]. Large mobility of the EL2 loop was observed in those MD studies that reported on the stability of the loop [44], [45]. This does not seem to be compatible with the structural requirements of the zinc binding site in DAT. RMSD values were reported to be larger than 0.4 nm, if reported. Our initial models were created while using the close proximity between residues H193, H375 and E396 as the only restraint. These models suffered from the same high mobility upon rigorous testing of EL2 in MD simulations and we found a wide range (0.4 to 1.4 nm) of different RMSD values between independent runs. The RMSD were calculated by first fitting the transporter to the hash domain and subsequently probing the mobility of EL2 by calculating the Cα RMSD value of residues 178 to 202. We observed that residue H193 did not remain in close proximity of residues H375 and E396. However, zinc binding induces a spatial constraint that requires immobility of the residues involved. We therefore refined our initial DAT homology model to include EL2 and accommodate the zinc binding site.

**Figure 4:**
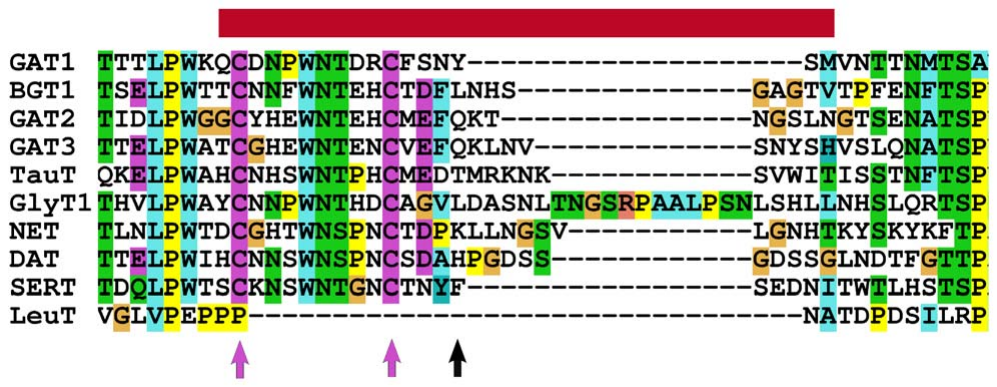
Sequence alignment of EL2. Sequence alignment of the EL2 region of the human NSS transporter aligned with the LeuT sequence, using clustal color coding. The EL2 region, for which the LeuT structure does not serve as a template, is indicated by a red bar. Residue H193 is indicated by a black arrow, the cysteines forming the disulfide bond are indicated by arrows colored in magenta.

**Figure 5:**
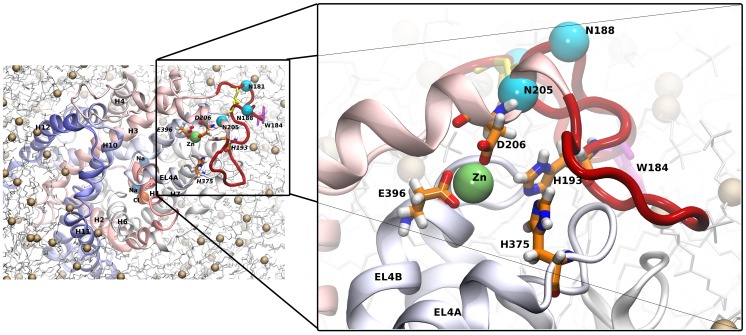
Model of the EL2. Structure of the membrane inserted DAT. EL2 is highlighted. The modeled loop is shown in dark red; the disulfide bridge formed by residues C180 and C189 in yellow and the Cα atoms of the glycosylated asparagines in blue. The membrane inserted side chain of W184 is depicted in magenta; the zinc coordinating residues are depicted in green.

Human NSS transporters possess a substantially longer EL2 than that found in the LeuT template (Fig. 4). We solved the conformational sampling problem by employing the high-affinity zinc binding site as a molecular ruler [18], [20]. Residues H375 and E396 are both located in EL4. They can be used to limit the possible spatial placement of H193. In addition, EL2 contains a conserved cysteine disulfide bond between C180 and C189. These two constraints allowed for a sharp reduction of the potential conformational phase space. The sequence alignment of all NSS transporters indicates that EL2 (indicated by the red bar in Fig. 4) consists of two parts with different properties, divided at residue H193 (indicated by a black arrow in Fig. 4). The first part, which precedes H193, is conserved in length and amino acid type and thus will most likely be structured. However, the second part, which is between H193 and the EL2-helix, is variable in length. It is also rich in glycine and serine and is therefore predicted to be largely unstructured. In an iterative approach, first 200 homology models were built and their quality was assessed by the MODELLER [60] energy function and the DOPE score [61]. The 10 best models were inserted into a POPC membrane and probed for stability by MD simulation (duration 50 ns) as described in “material and methods”. Structural restraints were imposed on loop modeling to reduce the search space during model building. These restraints were based on the following predictions: (i) the three N-glycosylation sites N181, N188, and N205 ought to be solvent exposed to allow for glycosylation [62], [63]; (ii) C180 and C189 form a disulfide bond and removal of this disulfide-bridge ablates surface expression [64]; (iii) H193 must be in close proximity to H375 and E396 [18], [20]: the zinc ion is coordinated by the side chains of all three residues, hence, H375 and E396 in EL4 served to constrain the position of H193. Assuming that the geometry of the EL2-helix resides above E396, two options were available to place H193: (i) placement above the two EL4 helices, or (ii) between H5, H7 and H8 at the edge of the transporter. The latter, in addition, establishes the transporter-membrane interface. The first option could be ruled out: residue conservation analysis did not allow to predict any conserved region in the EL2-helix. If the conserved part of the EL2 loop (from helix 3 to H193) would fold over and thereby interact with the EL2-helix, then the buried residues should be conserved in orthologous DAT sequences. Furthermore, residues with similar biophysical properties should be found in the closely related paralog NET, because the same zinc-binding site can readily be engineered onto NET [18]. In addition, a similar pattern would be expected in other members of the NSS family. We tested several models in which the loop was folded above the EL2-helix. In all these MD simulations, we observed an unstable loop structure. The zinc coordinating residue H193 moved away from the other two coordinating residues on the EL4 (H375 and E396) and we did not observe events where it would re-establish the contact. This is not in accordance with the high degree of conservation observed in the first part of EL2. If the loop was freely floating, there would be no evolutionary restrain that promoted the observed conservation. This would be incompatible with the requirements for zinc binding. The second option constrains the backbone of the EL2 behind the EL2-helix, in a similar manner to that seen in the recently published inward- and outward-facing structures of LeuT [5]. Here, H193 can be positioned between H375, E396 and the membrane, and hence above H5, H7 and H8 as shown in Fig. 5. This positioning allowed for EL2 to cover the hydrophobic surface of H5, H7, H8 and EL4B. Furthermore, EL2 shielded this covered hydrophobic part from the charges of adjacent lipid head groups. In support of this notion, simulations that started from this conformation revealed a much higher stability and resulted in a stable conformation after several iterations of model creation and testing.

The C180–C189 disulfide bond and the positioning of H193 greatly reduced the available phase space. Indeed, the sequence stretch before and after the disulfide bond had to be almost fully extended to reach from the end of helix H3 to residue H193 at the zinc binding site. The remaining amino acids between C180 and C189 form a loop. Its length is highly conserved within the NSS family (see magenta arrows in Fig. 4). Two residues in the center of this stretch (W184 and N185) are almost fully conserved among all NSS sequences. The other 6 amino acids are not conserved by residue but rather by length and type: they are either polar or charged. Such a high degree of conservation indicates an evolutionary constraint: tryptophanes are found in membrane proteins with high frequency at the hydrophobic/hydrophilic interface of membranes [65], [66]. A free energy minimum has been identified in this region for tryptophanes when they are moved from the water into the center of the membrane [67]. The tryptophan W184 could therefore act as a membrane anchor for the loop formed between C180 and C189 [68] (Fig. 5). In support of this notion, all simulations that started from a membrane-inserted W184 structure remained stable in the lipid-water interface. In contrary, this residue showed almost un-constrained movements in simulations where W184 was not inserted in the membrane. Hence, this membrane-association strongly restricts the movement of the entire loop. There is experimental evidence to support this model: if W184 is mutated to leucine, it abolishes cell surface expression of DAT consistent with the fact that it no longer shows a free energy minimum at the water-lipid interface [55].

**Figure 6:**
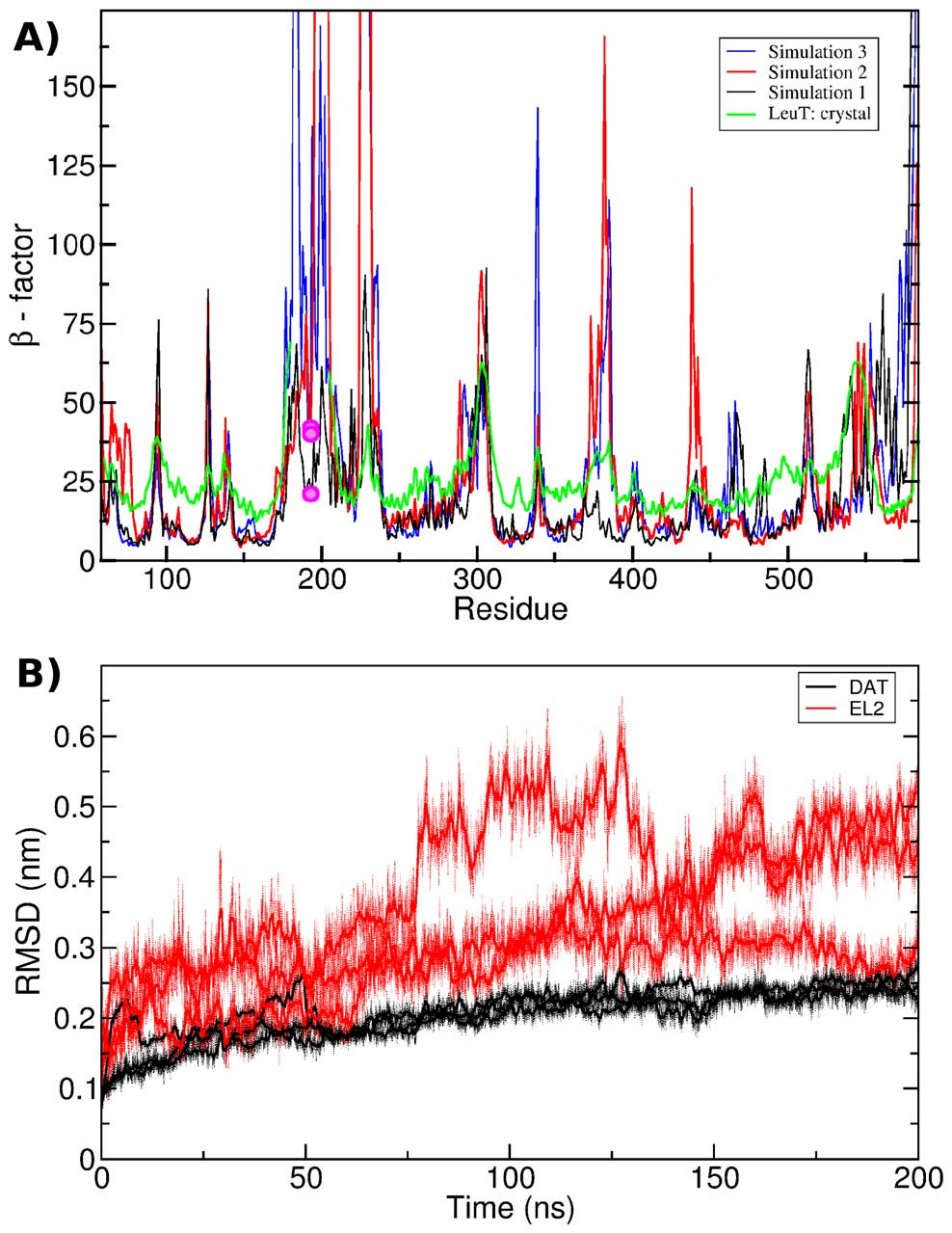
RMSF and RMSD values. A) Comparison of atom mobility with the β-factors of LeuT. The β-factors of the Cα atoms in DAT were calculated from the three 200 ns simulation. The position of H193 in the EL2 loop is indicated by a magenta circles. The regions of low mobility correspond to the transmembrane regions, while the connecting loops show greater flexibility. B) Time evolution of the RMSD. The RMSD value of the modeled EL2 (residue 178 to 202) is shown in red, the overall RMSD of DAT is shown in black.

Three models were challenged by 200 ns long MD simulations. The refined DAT model were stable over the entire length of the simulation as shown in Fig. 6. The overall RMSD of the DAT reached a plateau at 0.24–0.26 nm. This value is expected for proteins of 500 to 600 residues. In contrast, EL2 alone was slightly more mobile and leveled off at 0.3–0.5 nm (Fig. 6B). The increased flexibility of the EL2 loop reported here is to be expected, because loops are in general less restrained than the core of the transporter. We noted that the serine and glycine rich stretch of the EL2 loop between H193 and the EL2 helix (residue G195 to G203) moved freely and showed large motional amplitudes. This structural mobility was expected according to the primary sequence. The ß-factors show the mobility of DAT at a per-residue level (Fig. 6A). The pattern of higher and lower mobility mirrors the helical parts and the loop regions of the DAT structure (Fig. 6A). The position of H193 is indicated by circles colored in magenta. The restricted mobility of H193 is clearly visible, as is the large mobility of the serine and glycine rich stretch (residue G195 to G203). The observed difference in motions of the glycine rich stretch between the three simulations can be attributed to limited sampling. We observed larger ß-factors in the EL4A helix (residue K374 to D385) in two out of three simulation. We found a shift in the relative position of the helix giving rise to the large ß-factors, while the EL4A helix itself remained stable throughout the simulation.

**Figure 7:**
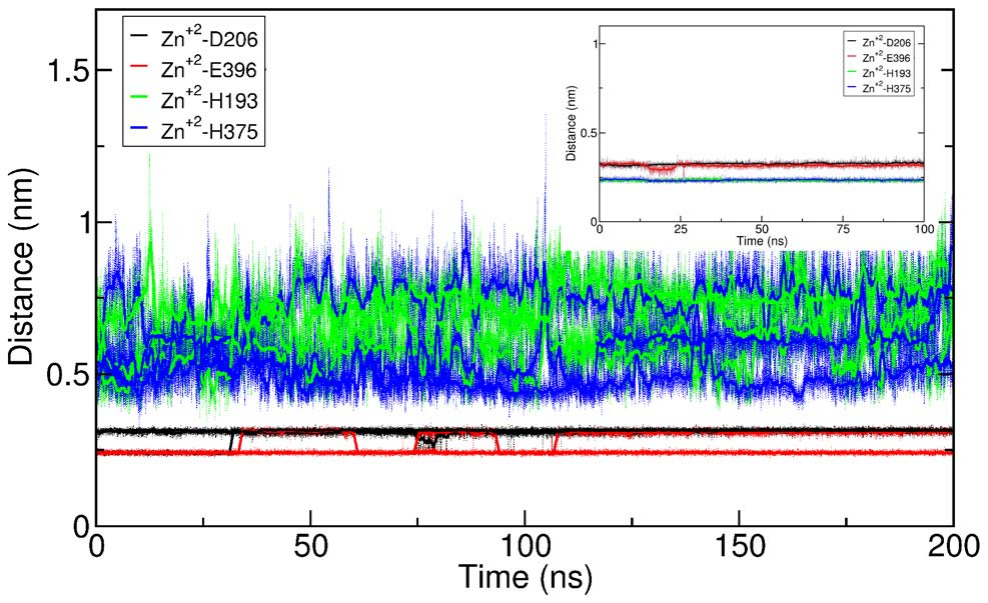
Distances between zinc and the coordination residues. The distances between the zinc atom and the coordinating residues are shown for the three 200 ns simulations. The carbon atom of the carboxy group were selected for D206 (Cγ) and E396 (Cδ) while the Nε nitrogen was chosen for H193 and H375. The insert in shows the time evolutons of the same distances from the trajectory where the zinc-histidine nitrogen interaction was defined as a chemical bond.

We observed stable zinc binding sites in all three simulations (see Fig. 7). Residue E396 shows a bi-dentate zinc coordination in two out of tree simulations, indicating a strong interactions between the bound zinc and the carboxyl group of the E396 side chain. The interaction of histidine with zinc has a chemical bond component and is geometrically restrained with a strong distance dependence. These quantum chemical effects are typically not correctly described by classical static forcefields including OPLS. We opted to maintain the full charge on the zinc ion and to not add any term to for histidine-zinc interaction. This choice was motivated by the aim of the study, as we intended to challenge the geometry of the zinc-binding site and not to enforce it. In this way, the structure can change, if it was unstable. The two histidine side chains of residues H193 and H375 do both show a larger distance to the zinc ion as compared to the charge-charge interaction between E396 and zinc, but the local geometry remains stable. The interaction of zinc with the histidine side chains is found to be a direct interaction or water bridged. The reason for this behavior can be found in the full charge of +2 on the zinc and the much smaller partial charge of the Nε atom of the histidine side chain. The zinc does therefore present a strong attraction force for solvent and water penetration must be expected. The crystal structures of zinc containing proteins reveal average zinc-to-nitrogen distances of ∼0.2 nm [69], [70]. This would cause an atomic overlap and strongly contribute to repulsion according to the Lennard-Jones non-bonding interaction term. A σ value equal to 0.252 nm marks the distance beyond which the Lennard-Jones potential of the OPLS force field of the zinc-to-nitrogen non-bonded interaction becomes attractive; the minimum of the potential is found at 0.33 nm. This strongly supports the nature of the zinc-histidine interaction as chemical bond. Despite this shortcoming, both histidine residues H193 and H375 remained firmly located in the vicinity of zinc. We expected thermal motions and water penetration to be a destabilizing force for the structure; therefore, it is remarkable that our models remain stable over the entire 200 ns period, further supporting the correctness of our model.

We re-parameterized the charges of zinc and the side chains of the coordinating residues H193, D206, H375 and E396 using “R.E.D. server” [71], [72] in order to correctly define the chemistry and test the model. “R.E.D. server” was originally developed to create OPLS type charges by fitting partial charges to quantum chemical calculations. We applied the same OPLS bonded parameter as used in a recent study of the zinc containing enzyme human carbonic anhydrase [73]. The distance between zinc and the Nε of the histidine side chain was maintained as parameterized in a 100 ns long simulation. The trajectory revealed that the distance to the two negatively charged residues D206 and E396 was unchanged since both residues remained in contact with the zinc ion (See insert in Fig. 7). RMSD as well as RMSF revealed that the addition of the chemical bonds did not affect the structure of the model or its dynamic behavior. In particular, the RMSD of the DAT reached the same level, while we observed that the RMSD of the EL2 loop became smaller, leveling off at 0.15–0.25 nm as compared to 0,2–0.3 nm observed without the zinc-nitrogen chemical bond.

### A fourth residue completes the zinc binding site

**Figure 8:**
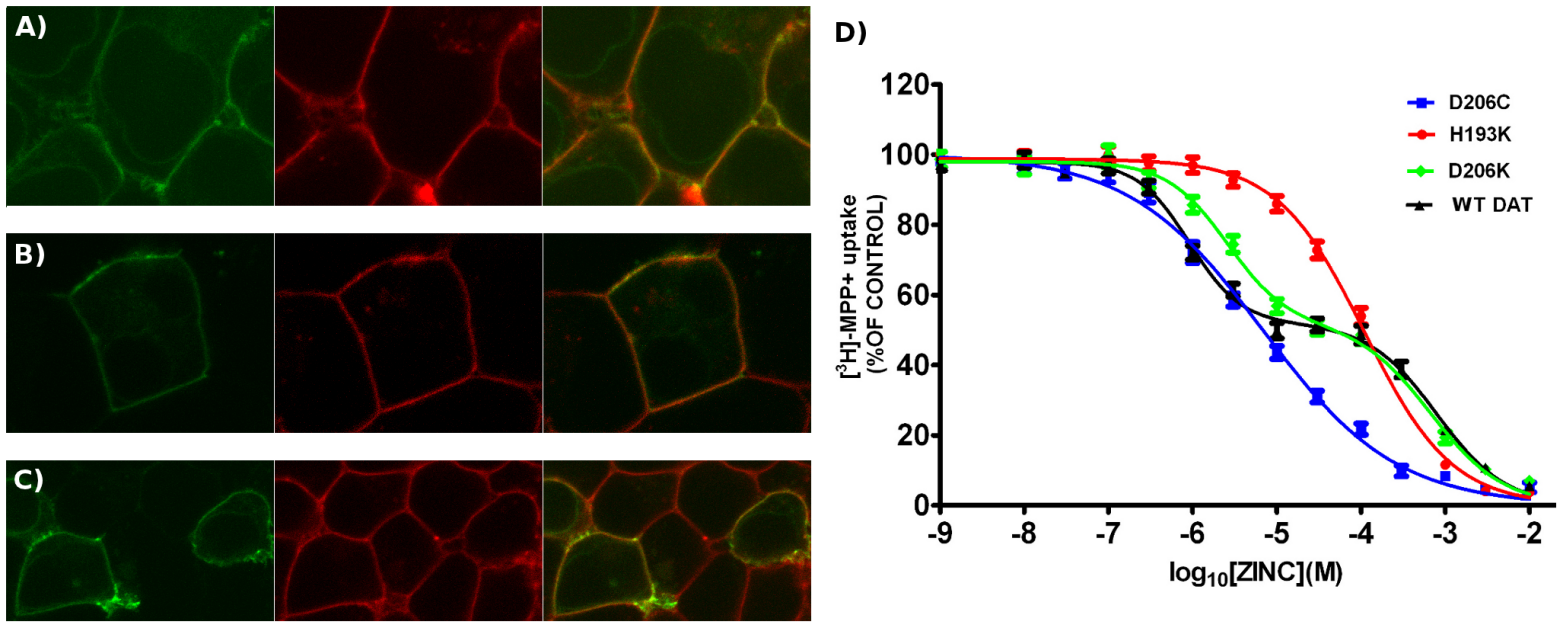
Wild-type and mutant DAT surface expression as well as inhibition of [^3^H]MPP^+^ uptake by zinc. Confocal images demonstrating the surface expression of A) wild-type DAT, B) DAT-D206K and C) DAT-D206C in transiently transfected HEK293 cells. The YFP-tagged transporter is shown on the left hand side in green, the plasma membrane is visualised by trypan blue staining in red. The merged view on the right hand side of each image highlights the co-localisation of both the wild-type and mutant transporters with the plasma membrane. D) Zinc inhibition of [^3^H]MPP^+^ uptake in HEK293 cells transiently expressing wild-type DAT (black), DAT-D206K (green), DAT-H193K (red) and DAT-D206C (blue). Data are expressed as percent of [^3^H]MPP^+^ uptake in the absense of zinc (control) for each transporter. This 100% control value amounted to 7.67±1.8 pmol/min/well. Data represent the mean ± S.E. of 6–8 experiments performed in triplicate.

Analysis of the endogenous high-affinity zinc binding site in DAT revealed that the first coordination sphere of zinc includes a previously unrecognised fourth residue: D206 (Fig. 5). This aspartate is present in all orthologous DAT sequences, which contain H193. In our simulations, D206 consistently interacted with zinc via its carboxyl moiety, thereby completing the first coordination shell of zinc. We found that the D206 – zinc distance displayed a stable time dependent behavior similar to the interaction of zinc with E396. The main difference to E396 is the coordination geometry. The carboxyl group of E396 mainly formed a bi-dentate coordination with both of its oxygens. In contrary, D206 coordinated zinc typically with only one oxygen atom, while the second oxygen of the carboxyl group interacted with the surrounding bulk water. If this model were true, then we would expect that modification of this residue should alter the affinity of extracellular zinc for DAT. We therefore tested this hypothesis by mutating D206 to lysine (DAT-D206K), to the zinc coordinating residues cysteine (DAT-D206C), histidine (DAT-D206H) as well as to glutamate (DAT-D206E) and alanine (DAT-D206A). HEK293 cells were transiently transfected with plasmids encoding either the wild-type or mutant versions of DAT. Mutants were expressed on the cell surface and were thus indistinguishable from YFP-DAT when examined by confocal microscopy, with the only exeption of the histidine mutant. The large, rigid and ingombruent size of the histidine side chain is probably responsible for a folding problem, ER retention and subsequent degradation. For clarity reasons we focus in the discussion of the results on the two mutants DAT-D206K and DAT-D206C and summarize transport inhibition data for all mutants in Table 1. The confocal microscopy images of wild type DAT and of the two mutants DAT-D206K and DAT-D206C are shown in Fig. 8A–C, indicating similar surface expression. The two mutants also transported the substrate [^3^H]MPP^+^ with rates (5.86±0.8 and 8.39±1.6 pmol/min/well for DAT-D206C and DAT-D206K, respectively) that were comparable to that of wild-type DAT (7.67±1.8 pmol/min/well). These values are within the range of previously published uptake rates for DAT expressed in heterologous cell systems [18], [21]. We assessed the ability of zinc to inhibit substrate uptake by these mutants and used wild-type DAT and DAT-H193K as reference: in wild-type DAT (black symbols in Fig. 8D), zinc exerted a biphasic inhibition on substrate uptake. The first component (IC50 = 1 µM, see Table 1) is due to interaction of zinc with its high-affinity binding site. The second component refects an action on low-affinity sites [18], [21]. Accordingly, in DAT-H193K, the affinity of zinc to the high-affinity site was lowered so much that it was not possible ot resolve the two components resulting in a homogeneous inhibition curve (Table 1). Substituting D206 by lysine reduced the inhibitory potency of zinc on the high-affinity component by approximately three fold (green symbols in Fig. 8D; Table 1). This reduction in inhibitory potency of zinc is not as large as that observed for the H193K mutant. Substitution of D206 by cysteine, which is known to be a zinc coordinating residue, also led to an apparent reduction in zinc binding affinity for DAT (Table 1). However, in DAT-D206C, zinc was a more effective blocker than in any other version of DAT (blue symbols in Fig. 8D). This resulted in almost complete inhibition of transport upon saturation of the high affinity site (99% occupancy predicted at 250 µM). Thus, inhibition by non-specific binding could no longer be observed. Mutation of D206 of glutamate or alanine did not result in any significant change in the inhibitory potency of zinc (see Table 1).

**Table 1:** IC_50_ values of [^3^H]MPP+ uptake inhibition by zinc.

	High-affinity site: IC_50_ (Zn^2+^) µM	Low-affinity site: IC_50_ (Zn^2+^) µM
DAT (wild-type)	0.91±0.1	798±84
DAT (D206C)	6.81±1.4	-
DAT (D206K)	2.54±0.8	682±100
DAT (D206A)	0.79±0.1	219±14
DAT (D206E)	1.22±0.3	857±76
DAT (H193K)	-	102±8

Inhibition of [^3^H]MPP^+^ uptake by zinc in HEK293 cells transiently expressing wild-type or mutant transporters. Inhibition curves were fitted to either a mono or bi-phasic model using Graphpad Prism version 4. IC_50_ values are given as mean ± S.E. of n = 3–8 experiments.

**Figure 9:**
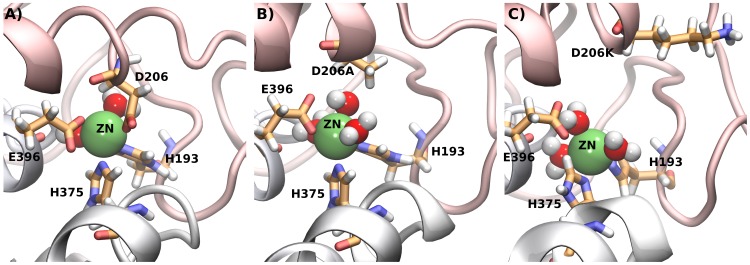
Water molecule interacting with zinc. A) The side chain of D206 does directly interact with zinc. A comparison with simulations of the B) D206A and the C) D206K mutants reveals that in both cases a water molecule takes the places of the carboxyl group of D206.

We created models of the D206K and the D206A mutants to investigate the effects of these mutations in more detail. The models included the zinc-nitrogen interactions defined as a chemical bond (see above). Simulations were carried out for 20 ns and remained stable for both mutants: the RMSD to the starting structure was 0.15 nm and we did not observe any conformational changes. The side chain of E396 remained in direct contact with the zinc ion as observed for wild type DAT. In the mutant D206A, a water molecule takes the place of the carboxyl group of aspartate 206 (Fig. 9) that interacts with zinc: the side chain of alanine is uncharged and small enough to leave the space. A water molecule can be observed in the same position in the D206K mutant: the long side chain of D206K is observed to extend into the solvent. Thereby, it offers similar space opportunities next to zinc as seen in D206A (Fig. 9). The positive charge on the lysine side chain does nevertheless interact with the zinc ion by long ranged electrostatic interactions. This repulsive force reduced the affinity for zinc as was observed in the uptake inhibition experiments (see Fig. 7).

## Discussion

Analysis of the human genome predicted that approximately 3000 proteins are zinc binding proteins [74]. These can be classified into two main groups based on the functional role of zinc: either enzymatic or structural [69]. Zinc accepts 4 to 6 ligands in its first coordination shell [70] and interacts with nitrogens provided by the imidazol ring of histidine, carboxyl oxygens supplied by aspartate or glutamate and sulfur atoms from the sufhydrdyl group of cysteine. The binding affinity of zinc is also controlled by the protein environment, with affinities in the picomolar range for intracellular proteins and micromolar for extracellular proteins. However, the binding affinity of zinc does not appear to depend on the specific nature of the interacting atoms, as very similar affinities have been observed with varying types of protein side chain ligands [70]. In almost all cases, if zinc is found in the active side of an enzyme, one of the coordination sites is not from a protein side chain: this allows the substrate to directly interact with the zinc atom of the enzyme. In contrast, structural zinc binding sites are characterized by a completed first coordination shell of zinc, with all coordinating atoms originating from protein side chains. Three zinc coordinating residues (H193, H375 and E396) have already been characterized in DAT [18], [20]. We identified a fourth residue (D206) in the first coordination shell of zinc: this is in line with the structural-regulatory and non-enzymatic function of the zinc binding site. The mutation of D206 to lysine reduced the affinity of DAT for zinc, but not to the same extent as seen in the H193K mutant. The positively charged Nζ atom of the H193K mutant side chain is expected to retain the same orientation in the mutant protein. It can therefore be assumed to be located in the center of the zinc binding site and to directly take the position of the zinc ion. In contrast, the positively charged Nζ atom of the D206K mutant should be oriented towards the solvent and does therefore not compete to the same extend with zinc for its interaction site. The substitution of the zinc coordinating residue D206 with the zinc ligand cysteine had a very different effect: it induced a change in the regulatory effect of zinc binding and had a only modest effect on the binding affinity of zinc. Thus, a complete inhibition of substrate uptake was observed in DAT-D206C upon zinc binding. A similar effect was observed for the DAT mutants E396H and T400C [20]. The residual transport activity of the wild-type DAT after zinc binding can be explained by the specific properties of the carboxyl side chain of D206 and E496. The carboxyl moiety of aspartate and glutamate side chains can coordinate zinc by one or both oxygens and this interaction is entirely electrostatic in nature. Both factors allow for a larger range of possible amino acid-zinc distances and orientations, which enables them to be structurally promiscuous. In contrast, the interaction between the cysteine sulfur and zinc is predominantly a charge-transfer interaction and restricts the possible distances to a narrow window. This strongly reduces the distance promiscuity of the cysteine mutant and also explains the complete zinc inhibition of dopamine transport seen with DAT-D206C, as compared to the partial zinc inhibition of transport seen in wild-type DAT. We observed lower affinity of zinc to the DAT-D206C mutant as compared to wild type. It is conceivable that the geometry at the zinc binding site is optimal for aspartate, but less so for cysteine. Side chain solvent exposure and reduced geometrical promiscuity of cysteine may therefore be responsible for the lower binding affinity. The zinc inhibition curve of the D206E mutant was virtually identical with wild type. This was an expected result, as structural analysis showed that D206 coordinates zinc laterally and some room is available for structural adaptation. The extention of the side chain of residue 206 by one methylen group in the D206E mutant does therefore not have any significant impact on this interaction.

The D206A mutation was phenotypically silent. Mutations of residues known to be in contact with zinc, but without accompanying affinity-changes, have been described previously for several proteins, including enzymes [75], zinc finger proteins [76], and receptors [77]. The finding is therefore not without precedent that the D206A mutation does not affect zinc binding within the error bars of our experiment. The mutation of aspartate to alanine reduces the size of the side chain of residue 206 to an extend that is large enough for a water molecule to take the place of the 206 side chain and cooridinate zinc, as observed in zinc-based enzymes. The correct modeling of EL2, which harbors the zinc coordinating residue H193, is required to fully understand the functional impact of zinc on the DAT transport cycle. Up to now, molecular models of this extracellular loop have been established [31]–[35], [41], [44], [45], [47], but they have been rarely scrutinized. The EL2 of the NSS is 21 amino acids longer than EL2 in LeuT and thus the potential conformational space is prohibitively large for *ab initio* modeling. The dopamine transporter, however, provides an opportunity to reduce the number of possible configurations, as the loop can be restrained by the coordinates of the high-affinity zinc binding site. We traced this loop behind the EL2-helix, where it interfaces with the membrane. We observed that the loop is most restrained in its flexibility at position H193. This feature is very likely to be important for the transport reaction, because this region shows structural changes during the transport cycle, highlighted in recent structures of LeuT in the inward- and outward-facing conformations [5]. Here, helix H8 is part of the hash domain and H7 is part of the bundle, while helix EL4A and H2 partially follow the movement of the bundle. The mobility of the EL2-helix is facilitated by the conserved residue P213 within the EL2-helix that allows for helix bending. We observed that the sequence stretch between H193 and the helix of EL2 is very flexible, as indicated by initial analysis of NSS sequences. This high flexibility is most likely necessary to avoid any energetic barrier for transport.

Partial digestion experiments of rDAT, in which the second part of the EL2 loop (R218 in rDAT, located at the end of the EL2 helix and potentially R227 in the loop following the 2EL helix) is cleaved by trypsin [78] showed no difference in trypsin digestion upon zinc or substrate binding, while binding of different inhibitors, including cocaine and CFT, restrained the transporter in a state that showed decreased protease sensitivity. Identical proteolysis behavior with and without zinc suggests that zinc binding per se does not induce major changes in conformation of the EL2 loop. We can only speculate on the structural origin of the change in sensitivity to trypsin in light of the new inward- and outward-facing template LeuT structure: the LeuT structures show that the EL2 helix partially follows the movement of the bundle domain during the transport cycle. The main trypsin cleavage site was observed at the remote end of this helix. Inhibitors restrain the DAT in a single conformation, while in other conditions this was not necessarily the case. It is therefore conceivably that the trypsin sensitive conformation is not accessible in the inhibitor bound state.

**Figure 10:**
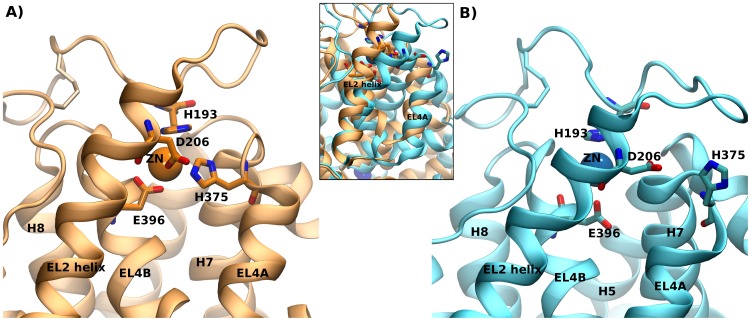
Outward- and inward-facing model of the zinc binding site. Models of the zinc binding site are shown that combine our model of the EL2 with the outward- (A) and inward-facing (B) structure of LeuT [5]. The insert shows an overlay of the two models after fitting them on the hash domain. The four zinc coordinating residues H193, D206, H375 and E396 are highlighted. A complete first coordination shell of zinc is observed in the outward-facing state (A), while in the inward-facing state (B), the first coordination shell is disrupted due to the translation of H375.

In functional terms, it has been shown that zinc binding promotes the population of the outward-facing conformation [79]. Structural analyses of the residues coordinating zinc revealed that H193 and E396 can be assigned to the hash domain. H375 is found on top of helix 7 and is therefore part of the bundle domain, while D206 is located on the helix in EL2 which partially follows the motion of the bundle. The model of substrate transport by NSS [5], [9] proposes that the bundle domain changes its conformation during transport: this movement closes the outer vestibule at the extracellular face of the transporter and opens the inner vestibule. Fig. 10 shows a model of the DAT zinc binding site, built by combining our EL2 loop model with the recent open-outward- (Fig. 10A) or inward-facing conformations (Fig. 10B) of the LeuT [5]. These models visualize the expected conformational change in the DAT and display the change in the first coordination shell of zinc upon transition from the outward- to the inward-facing conformation. The models uncovered that the rotational-translational movement of the bundle domain alters the geometry of the zinc binding site. The position of H375 was thereby displaced by 0.85 nm from the tetrahedral zinc coordinating geometry and was moved almost behind D206.

Hence, the first zinc coordination shell was disrupted. These observations allowed us to propose a two-step model of the mode of action of zinc on DAT: (i) the zinc ion is attracted to the binding site by the two negative charges of D206 and E396 and (ii) the tetrahedral coordination is completed by H193 and H375. Hence, binding of zinc selects for the outward-facing conformation, as the coordination by H375 is only possible in this conformation.

The strong phenotypes observed for the H193 and H375 mutants and the weaker effects of the D206 mutants can be rationalized in the context of this conformational change. Inhibition of transport by zinc is observed, because the bound zinc ion puts a restraint on the distance between H375 on the moving bundle domain and H193 plus E396 on the hash or scaffold domain. A mutation on either side abrogates this tight restraint that reduces the transport rate of DAT by ∼50% upon zinc binding. As a consequence, inhibition of transport cannot be observed in the experimental assay even if zinc still binds: this is the result of the mutation of the essential interaction site on one of the two domains. The situation for D206 is different: residue D206 is located within the helix of the second extracellular loop. This helix does partially follow the motion of the bundle domain (see Fig. 9). Because the helix is not strictly associated with either domain, it is not directly involved in the essential interaction that changes turnover number of DAT upon zinc-binding. In this sense, D206 has a supportive role, while H193, H375 and E396 are essential for controlling transport rates.

## Materials and Methods

### Modeling

The alignment was created using muscle [80] and is based on the alignment published by Beuming et al. [40]. The alignment was modified in EL4 by closing the gaps before and after the EL4A helix (See Fig. S1). We thereby obtained improved clustal quality scores. The structure of LeuT with the PDB ID: 2A65 [7] was used as the reference template for creating the homology model of the human dopamine transporter. In addition to the LeuT template, parts of the best structure from the previous model building interaction were used as templates in subsequent iterations of model building, optimization and testing. MODELLER version 9.8 [81] was used to create 200 structures using the automodel procedure. The structures were evaluated using the MODELLER energy score and the DOPE score [61]. The best models were further evaluated for compactness, accessibility of N-glycosylation site and geometry of the zinc binding site. The positions of the sodium ions in the LeuT structure were maintained in the DAT model. The chloride ion was placed as proposed in the literature [11]–[14].

### Simulations

The 10 best models were inserted into a system consisting of a pre-equilibrated membrane created to harbor the dopamine transporter, plus water and counter ions. In order to prevent atom overlaps, the g_membed method [82] was used to insert the human DAT model. After equilibration of the surrounding environment for 2.5 ns, position restraints on DAT were slowly reduced in 4 steps, applying 1000, 100, 10, and 1 kJ/mol, respectively, each time simulating for 2.5 ns. Distance restraints designed to maintain the secondary structure were in addition to the position restraints applied using a force constant of 100 kJ/mol. An additional 20 ns equilibration simulation was carried out, where distance restraints on the secondary structure were maintained while position restraints were removed. The stability of the model was further challenged in a final 20 ns unconstrained MD. In total 15 iterations were necessary in order to identify good models that were stable in MD simulations. The best model was further challenged in a 200 ns simulation.

MD simulations were carried out using the GROMACS 4.5.3 MD package [83], [84], applying the OPLS force field [85], [86]. The 1-palmitoyl-2-oleoyl-sn-glycero-3-phosphatidylcholine (POPC) lipids of the membrane were represented by Berger lipids, [87] converted into the format of the OPLS all atom force field by following the procedure proposed by Neale (http://www.pomeslab.com/files/lipidCombinationRules.pdf). The water was represented as SPC water [88]. The simulations were carried out at a constant temperature of 310 K, using the v-rescale (τ = 0.1 ps) thermostat [89], while coupling the protein, the membrane, and the water/ions separately. The pressure was maintained at 1 bar using the weak coupling algorithm [90] with a coupling constant of 1.0 ps and a compressibility of 4.5×10−5 bar-1. The electrostatic interactions were evaluated using the smooth particle mesh Ewald method [91], with a cutoff of 1.0 nm. The long-range electrostatic interactions were calculated with fourth-order B-spline interpolation and a Fourier spacing of 0.14 nm. The Lennard-Jones interactions were evaluated using a cutoff of 1.0 nm with the neighbor search list updated every ten steps. Long range correction for energy and pressure were applied. Bonds and angles of the water molecules were constrained using the SETTLE algorithm [92], while all other bonds were constrained using LINCS [93].

### Parameterization of the zinc binding site

The coordinates of zinc and the side chains of the four coordinating residues (the imidazol rings of the two histidines, and the carboxyl groups of aspartate and glutamate) were extracted from our homology model. A methyl group was added where the chemical bonds were truncated. The geometry was first optimized at the semi-empirical level and then at the quantum-chemical level using Gaussian through the R.E.D Server [71], [72]. Partial charges are subsequently derived by the R.E.D Server that are tailored for the OPLS force field based on the quantum chemical calculations. The partial charges were then adjusted to account for the already present partial charges that were already present on these amino acids to obtain an overall unit charge.

### Chemicals

[^3^H]1-methyl-4-phenylpyridinium ([^3^H]MPP^+^; 85 Ci/mmol) was supplied by American Radiolabeled Chemicals (St. Louis, MO). Chemicals at analytical grade were obtained from Sigma Aldrich. Cell culture media, and antibiotics were obtained from Invitrogen.

### Plasmids

Wild-type human DAT was generously donated by Marc G. Caron [94]; DAT H193K was a generous gift from Ulrik Gether [18]. Wild-type and mutant DAT were YFP tagged at their N-terminus as described in Egana et al. [95]. Mutagenesis was performed using the Quickchange Lightning Kit by Agilent Technologies. Primers were designed using the “quickchange primer design tool” by Agilent. Primers used were: D206K: (5′-3′): CAGCTCGGGCCTCAACAAGACTTTTGGGACCACAC; D206C: (5′-3′): CAGCTCGGGCCTCAACTGCACTTTTGGGACCACA.

### Cell culture and transfections

HEK293 cells were cultured in Dulbecco's modified Eagle's medium (DMEM) with high glucose (4.5 g/liter) and L-glutamine (584 mg/liter), supplemented with 10% fetal calf serum (FCS), 100 units/ml penicillin and 100 µg/ml streptomycin. The cells were maintained at 37°C in a humidified atmosphere of 5% CO_2_/95% air on standard plastic culture ware. The cells were transiently transfected with 2–10 µg of plasmid DNA as required, using the calcium phosphate precipitation method.

### Confocal microscopy

Cells were seeded onto poly-D-lysine coated 15 mm coverslips for confocal microscopy twenty-four hours after calcium phosphate transfection. Forty-eight hours after transfection, the cells were analysed by confocal microscopy. The plasma membrane was identified using 0.025% trypan blue as previously described [96]. All images were acquired using a Zeiss Axiovert LSM510 confocal laser-scanning microscope.

### Uptake assays

Cells were seeded at 0.5×10^5^ cells/ml into poly-D-lysine coated 48 well plates twenty-four hours after calcium phosphate transfection with either wild-type or mutant DAT. Uptakes were performed as described previously [21]. In brief, the cell medium was aspirated and the cells were washed once with Krebs-HEPES buffer at room temperature. The washed cells were pre-incubated with Krebs-HEPES buffer in the presence of 0–10 mM ZnCl2 for 5 min at room temperature. In the first step of the assay, this buffer was replaced with Krebs-Hepes buffer containing the appropriate ZnCl2 concentration and 0.02 µM [^3^H]MPP^+^. After three minutes substrate uptake was stopped by exchange of the substrate containing buffer with ice-cold Krebs-Hepes buffer. The cells were lysed in 1% SDS and transferred to scintillation vials. Scintillation cocktail was added and the vials were assayed for [^3^H] content by liquid scintillation counting. Non-specific uptake was determined in the presence of 10 µM mazindol.

### Data analysis

Uptake data are given by mean values ± S.E, obtained from n = 6–8 experiments. Total uptake data were corrected for non-specific uptake and expressed as a percentage of 0 mM ZnCl_2_ for each experiment. IC_50_ values were calculated for each mutant by performing non-linear regression analysis using PRISM version 4.0 (GraphPad Software, Inc., San Diego, CA).

## Supporting Information

Figure S1 The LeuT and DAT sequence alignment used for model creation.
(TIFF)
