# Supplementary figures and images for: Mutational Analysis of the High-Affinity Zinc Binding Site Validates a Refined Human Dopamine Transporter Homology Model
Source: PLoS Comput Biol. 2013 Feb 21;9(2):e1002909. doi: 10.1371/journal.pcbi.1002909 (PMC3578762; doi:10.1371/journal.pcbi.1002909)

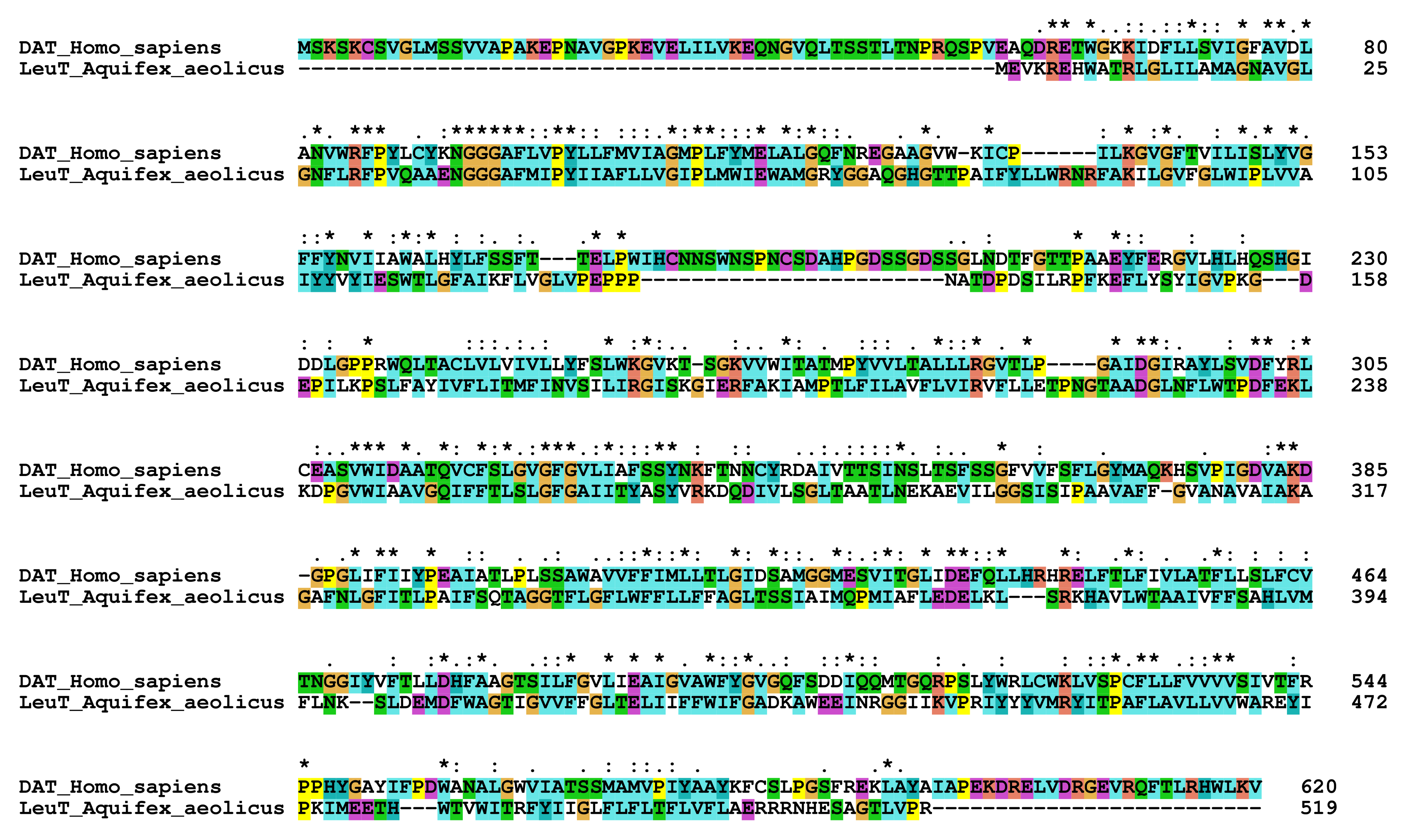

Supplement: Figure S1 — The LeuT and DAT sequence alignment used for model creation. (TIFF) [file pcbi.1002909.s001.tiff]
